# Mutation of epigenetic regulators TET2 and MLL3 in patients with HTLV-I-induced acute adult T-cell leukemia

**DOI:** 10.1186/s12943-016-0500-z

**Published:** 2016-02-16

**Authors:** Chien-Hung Yeh, Xue Tao Bai, Ramona Moles, Lee Ratner, Thomas A. Waldmann, Watanabe Toshiki, Christophe Nicot

**Affiliations:** Department of Pathology, Center for Viral Oncology, University of Kansas Medical Center, 3901 Rainbow Boulevard, Kansas City, KS 66160 USA; Department of Medicine, Division of Molecular Oncology, Washington University School of Medicine, Saint Louis, MO 63110 USA; Lymphoid Malignancies Branch, Center for Cancer Research, National Institutes of Health, Building 10, Room 4 N/115, 10 Center Drive, Bethesda, MD 20892 USA; Department of Medical Genome Sciences, University of Tokyo, Tokyo, Japan

**Keywords:** HTLV-I, Leukemia, ATL, LOH, TET2, MLL3, Epigenetic

## Abstract

**Background:**

Epigenetic regulators play a critical role in the maintenance of specific chromatin domains in an active or repressed state. Disruption of epigenetic regulatory mechanisms is widespread in cancer cells and largely contributes to the transformation process through active repression of tumor suppressor genes. While mutations of epigenetic regulators have been reported in various lymphoid malignancies and solid cancers, mutation of these genes in HTLV-I-associated T-cell leukemia has not been investigated.

**Method:**

Here we used whole genome next generation sequencing (NGS) of uncultured freshly isolated ATL samples and identified the presence of mutations in SUZ12, DNMT1, DNMT3A, DNMT3B, TET1, TET2, IDH1, IDH2, MLL, MLL2, MLL3 and MLL4.

**Results:**

TET2 was the most frequently mutated gene, occurring in 32 % (10/31) of ATL samples analyzed. Interestingly, NGS revealed nonsense mutations accompanied by loss of heterozygosity (LOH) in TET2 and MLL3, which was further confirmed by cloning and direct sequencing of DNA from uncultured cells. Finally, direct sequencing of matched control and tumor samples revealed that TET2 mutation was present only in ATL tumor cells.

**Conclusions:**

Our results suggest that inactivation of MLL3 and TET2 may play an important role in the tumorigenesis process of HTLV-I-induced ATL.

**Electronic supplementary material:**

The online version of this article (doi:10.1186/s12943-016-0500-z) contains supplementary material, which is available to authorized users.

## Background

Human T-cell leukemia virus type I (HTLV-I) is associated with fatal lymphoproliferative disorders known as adult T-cell leukemia/lymphoma (ATL) [1, 2]. The disease is classified into distinct subtypes - smoldering, chronic, acute and lymphoma - that differ in their clinical presentation and in their response to treatment [3]. Since the clinical subtypes of ATL have distinct genomic alterations and different clinical progression, these diseases require a different approach for treatment [4]. However, current therapies for ATL do not result in long-term remission and even the clinically less aggressive forms ultimately evolve to the acute. The 4 year survival rate for acute-, lymphoma-, chronic- and smoldering-type ATL is 11, 16, 36, and 52 %, respectively [5, 6]. The viral oncoprotein Tax plays an important role in initiation of events leading to cellular transformation [7, 8]. However, the fact that the disease has a low penetrance and is observed after a long latency period of several decades has led to the hypothesis that the virus initiates oncogenic events but is not sufficient for cellular transformation [9, 10]. In support of this notion epigenetic alterations are required for the development of ATL. Promoter hyper-methylation associated with loss of SHP1 expression coincides with the IL-2-independent transformation of T cells by HTLV-I in vitro [11]. SHP1 is one of the most frequently altered genes in ATL patients, with an overall hyper-methylation rate of 90 % [12]; other tumor suppressor genes inactivated by methylation in ATL include p53-related p73, CDKN2A and p21CIP1/WAF1 [13]. The fact that histone methyl-transferase EZH2 has been demonstrated to repress p57KIP2 expression through histone H3 lysine 27 trimethylation (H3K27me3) [14], and that p57KIP2 is methylated in nearly 50 % of newly diagnosed ALL patients [15], prompted us to analyze the status of cellular genes involved in chromatin silencing. In this study we use next generation sequencing (NGS) to characterize the genetic mutations in EZH1, EZH2, EED, SUZ12, DNMT1, DNMT3A, DNMT3B, TET1, TET2, TET3, IDH1/2, MLL, MLL2, MLL3, MLL4 and ASXL1. Our study revealed a high frequency of mutation in epigenetic regulators in ATL samples, suggesting that chromatin remodeling by some of these genes may play a role in the pathogenesis of ATL.

## Methods

### ATL patient samples

**Fig. 1:**
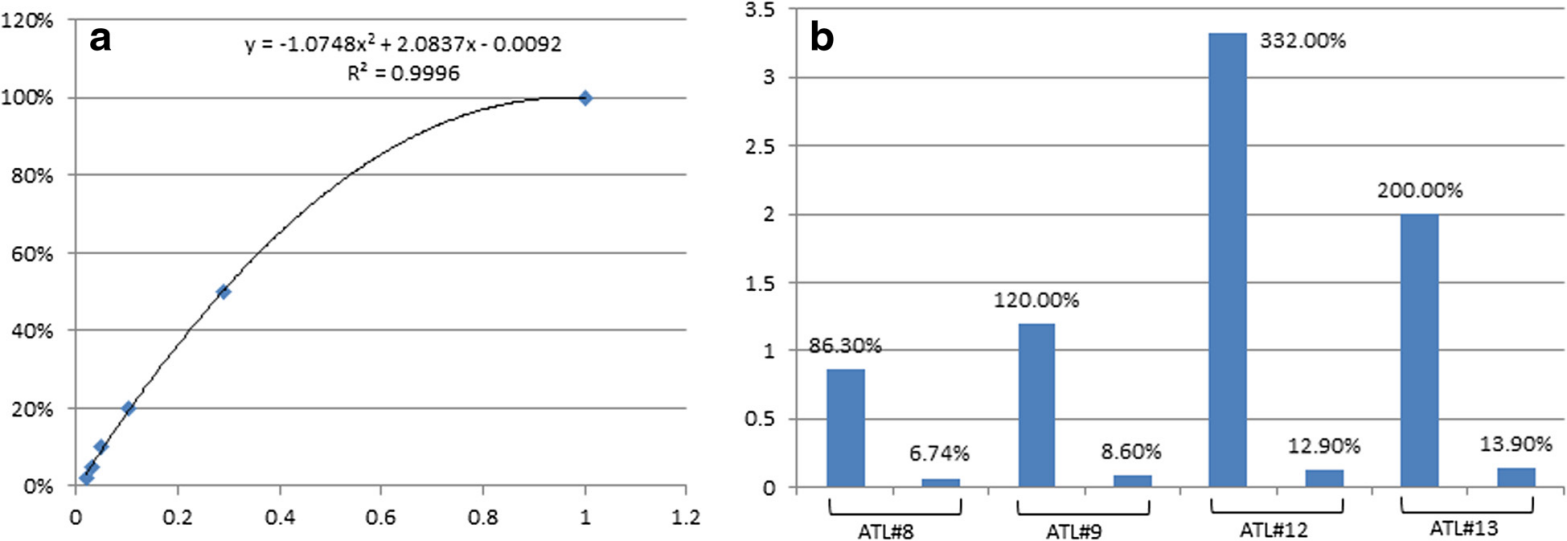
**a** A set of standard samples was prepared through dilution of HTLV-I transformed cell line DNA (TL) containing a single copy of integrated HTLV-I with HTLV-I negative 293 T cell DNA. Real time PCR was performed with 100 ng of mixed DNA. Both GAPDH and gag were detected and ΔCt was calculated by Ct gag - Ct GAPDH. The standard curve was created with ΔCt (X) and the percentage of HTLV-I viral load relative to TL cell (Y). **b** Proviral load was calculated in DNA samples isolated from high grade ATL lymphoma and matched-control B cells by real time PCR. Relative proviral loads were calculated using the standard curve established above

All patient samples were obtained after informed consent was provided and in agreement with regulations for the protection of human subjects according to the National Institutes of Health (NIH) guidelines. As for the samples from the Japanese material bank, they were provided from the biomaterial bank of the Japanese nationwide cohort study (Joint Study of Predisposing Factors for ATL Development, JSPFAD) that is approved by the ethical committee of the University of Tokyo (No. 14-15, No. 07-07 and No. 10-50). Genomic DNA was extracted using DNAZol (Invitrogen) from uncultured acute and lymphoma ATL samples. DNA samples 1–7, 10 and 11 were isolated from patients diagnosed with acute ATL. DNA samples 8, 9, 12 and 13 were isolated from patients with lymphoma ATL. HTLV-I proviral load was calculated by TaqMan real time PCR and compared with a standard curve established using C91PL HTLV-I transformed cell line harboring one proviral copy (Fig.1a). High tumor grade lymph node biopsy was used for ATL lymphoma patients as confirmed by real time PCR compared with B cells isolated from matched patient (Fig.1b).

### Next generation sequencing (NGS)

Exome Sequencing was performed by Perkin Elmer. DNA samples were evaluated using an e-gel and PicoGreen fluorometry to measure quality and quantity, respectively. DNA samples were then physically sheared to the desired size using a Covaris E220 Focused-ultrasonicator. Library preparation and enrichment were carried out using an Agilent SureSelectXT All Exon V3 kit and an automated sample preparation method derived from the manufacturer’s protocol. All subsequent steps were based on sequencing by Next Generation Sequencing methods on the Illumina Hiseq 2000 platform. Basecall files (*.bcl) were generated by the Illumina instruments and de-multiplexed and converted to fastq.gz format using CASAVA v1.8.2. Each pair of fastq.gz files was then aligned against human reference build 37 using BWA, v0.6.2. The resulting SAM files were converted to BAM format, sorted and indexed using SamTools v0.1.18. Duplicate reads in the sorted BAM file were marked using PicardTools v1.86. The duplicate marked BAM files were processed using GATK v1.6–13, following their “Best-Practices V3”. Each BAM was realigned around known INDELs and base quality scores were recalibrated, resulting in a recalibrated BAM file. Variants for each recalibrated BAM file were called using GATK Unified Genotyper, with SNPs and INDELs saved to separate files. These files were then hard filtered.

### Direct PCR-sequencing and TA cloning for analyses

Direct sequencing of ATL DNA was performed after PCR amplification using specific primers described below. In the case of MLL3 primers were located in introns surrounding exon 16. Primers amplify genome sequence from 152235498 to 152236241 of chromosome seven which encompass the MLL3 Exon16 nucleotide 2653 to 2769 (amino acid 884 to 923). MLL3 F: CAGGCTATAGTTGTTGTCGTCACCAAG; MLL3 R: CATAACATGATAGTAAGCAAATATCTATC. TET2-414 primers amplify nucleotide 842 to 1379 exon1 of TET2, which correspond to TET2 amino acid 281 to 459. TET2 Q414-F: ACTCTGAGCTGCCTCCAAAG; TET2 Q414-R: GAAGGTGGTGCCTCAGGTTT. TET2-876 primers amplify nucleotide 2403 to 2866 exon1 of TET2, which correspond to amino acid 801 to 955. TET2 Q876-F: TGTCCAAATGGGACTGGAGG; TET2 Q876-R: GATGCCACCTTAGAGCAGCA. Individual clones were obtained by TA-cloning (Invitrogen) and five clones were sequenced.

## Results

Despite profound epigenetic alterations in the genome of ATL cells, the genetic status of chromatin modifiers has not been investigated. In this study we performed next generation exome sequencing (NGS) to identify novel mutations in epigenetic regulators in ATL samples. The polycomb repressive complex 2 (PRC2) has histone methyltransferase activity and primarily trimethylates histone H3 on lysine 27 (H3K27me3), a mark of transcriptionally silent chromatin [16]. The PRC2 complex has four subunits: SUZ12, EED, EZH1 and EZH2. LOH mutations of EZH2 or SUZ12 have been reported in 25 % of T-ALL [17]. In addition, the loss of PRC2 activity cooperates with mutated Notch1 by allowing recruitment of the intracellular domain of Notch onto the promoter of target genes [17]. Along these lines, activated Notch is also required for ATL cell growth and tumor formation in an ATL mouse model [18]. While the EZH2 gene was not mutated in our study, 1/13 ATL sample had a mutation in SUZ12 (Additional file 1: Table S1). It will be interesting in future studies to investigate if there is any cooperation of EZH2 and/or SUZ12 with activated Notch in a larger cohort of acute ATL patients. The coding sequence of the other two subunits, EED and EZH1, was not mutated in any of the ATL samples tested. The possibility that some members of PRC2 may be regulated post-transcriptionally by microRNA or LncRNA in ATL cells is under investigation. Similarly, miR101, miR-26 and miR208b have been shown to target EZH2, miR-323-3p to target EED, and miR-200b to target SUZ12. An earlier study demonstrated that decreased expression of miR-101, but not MiR-26b, in acute ATL is in part responsible for elevated expression of EZH2 in these cells [19]. Consequently, increased expression of the EZH2 protein induced the silencing of miR-31, resulting in NIK-mediated activation of NF-kB in ATL cells [20]. Additional sex combs like transcriptional regulator 1 (ASXL1) interact with PRC2 and are likely involved in a cross-talk between chromatin silencing systems, PRC1/PRC2, the HP1α/CBX5 heterochromatin repressive complex and the polycomb repressive deubiquitinase (PR-DUB) complex. Mutation of ASXL1 has been reported in AML and chronic myelomonocytic leukemia (CMML) patients [21]. Our study revealed mutations of ASXL1 in 2/13 ATL samples (Additional file 1: Table S1). Interestingly, an ASXL1 somatic mutation, V1092M, detected in one ATL patient has also been reported in myeloproliferative neoplasms (MPN) and myelodysplastic syndromes (MDS) [22].

We next analyzed DNA (cytosine-5)-methyltransferases (DNMT1, DNMT3A and DNMT3B), which catalyze the transfer of methyl groups to specific CpG islands in DNA and are involved in maintenance or de novo methylation. Somatic mutations in DNMT3A have been reported as nonsense, frameshift, and missense mutations throughout the open-reading frame in 5–20 % of AML and MDS [23]. These studies suggested a potential gain-of-function that did not require the presence of a wild type copy of DNMT3A for altered function. Our analyses identified mutations in 7.5 % (1/13) of DNMT1 (isoform a) and DNMT3A (isoform b) and 15 % (2/13) of DNMT3B (isoform 1) of ATL samples. Interestingly, the same mutation at position N442K of DNMT3B was identified in two different unrelated ATL patients and has been reported in prostate cancer cells and the Cosmic Database.

**Fig. 2:**
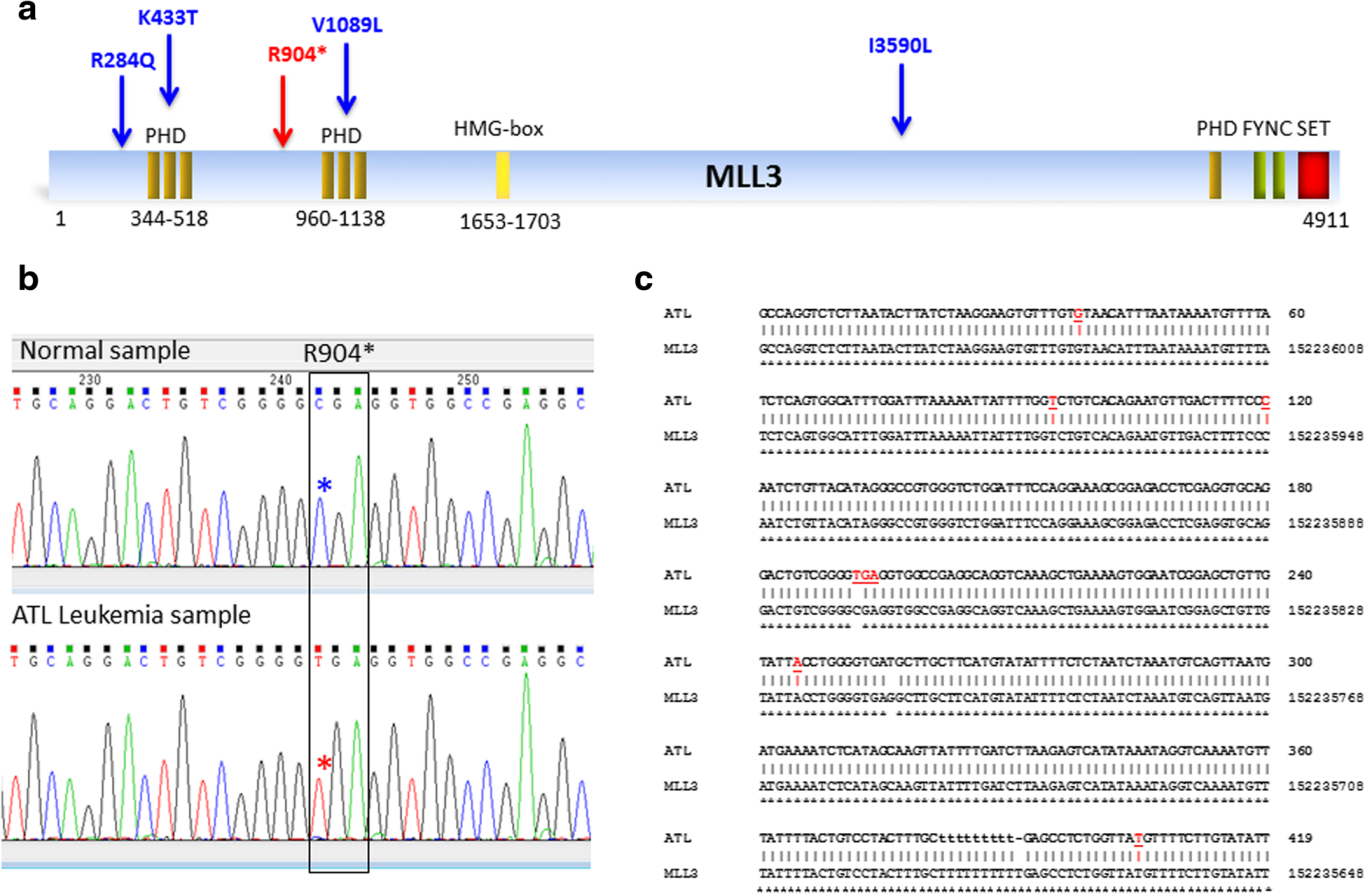
**a** Schematic representation of the MLL3 protein and distribution of mutations found in ATL patients. Nonsense and missense mutations found in ATL patients are shown in red and blue, respectively. **b** Chromatogram of the sequence of MLL3 from normal PBMC DNA (*top*) and MLL3 from ATL DNA with amino acid 904 nonsense mutation (*bottom*). **c** Alignment of ATL patient DNA KOE with MLL3 demonstrates the presence of a stop codon TGA in position 904. SNPs identifying the MLL3 sequence (different from Chr13) are labeled in red

The Mixed Lineage Leukemia (MLL) family of genes (also known as lysine (K)-specific methyltransferases (KMT2)) plays an important role in histone methylation and transcriptional activation and is involved as a regulator of growth of hematopoietic precursor cells. Mutation of MLL and MLL2 was observed in 7.5 % (1/13) of ATL patients. The MLL3 gene, which encodes a component of a histone H3 lysine 4 methyltransferase complex named the ASC-2- and Mll3-containing complex (ASCOM), has been implicated as a tumor suppressor gene due to its frequent mutations in multiple types of human tumors [24]. Exome sequencing has recently been used to identify an MLL3 germ line mutation in a pedigree of colorectal cancer and acute myeloid leukemia [25]. Mutations and LOH in MLL3 has been reported in various human cancers [26]. Our initial NGS analyses identified a high rate of nonsense mutations in MLL3 at position R904* of ATL samples (Fig.2a). This was interesting because early termination of MLL3 is predicted to produce a dominant negative form with oncogenic activities [27]. The presence of R904* on a highly conserved sequence homologous to MLL3 present on chromosome 13 likely contributed to the wrong assignment of a snp (rs200662726) in position R904* of the MLL3 gene in the NCBI database (Additional file 2: Table S2). Nevertheless, direct sequencing for all ATL DNA samples confirmed LOH for MLL3 in one ATL patient (Fig.2b and c).

**Fig. 3:**
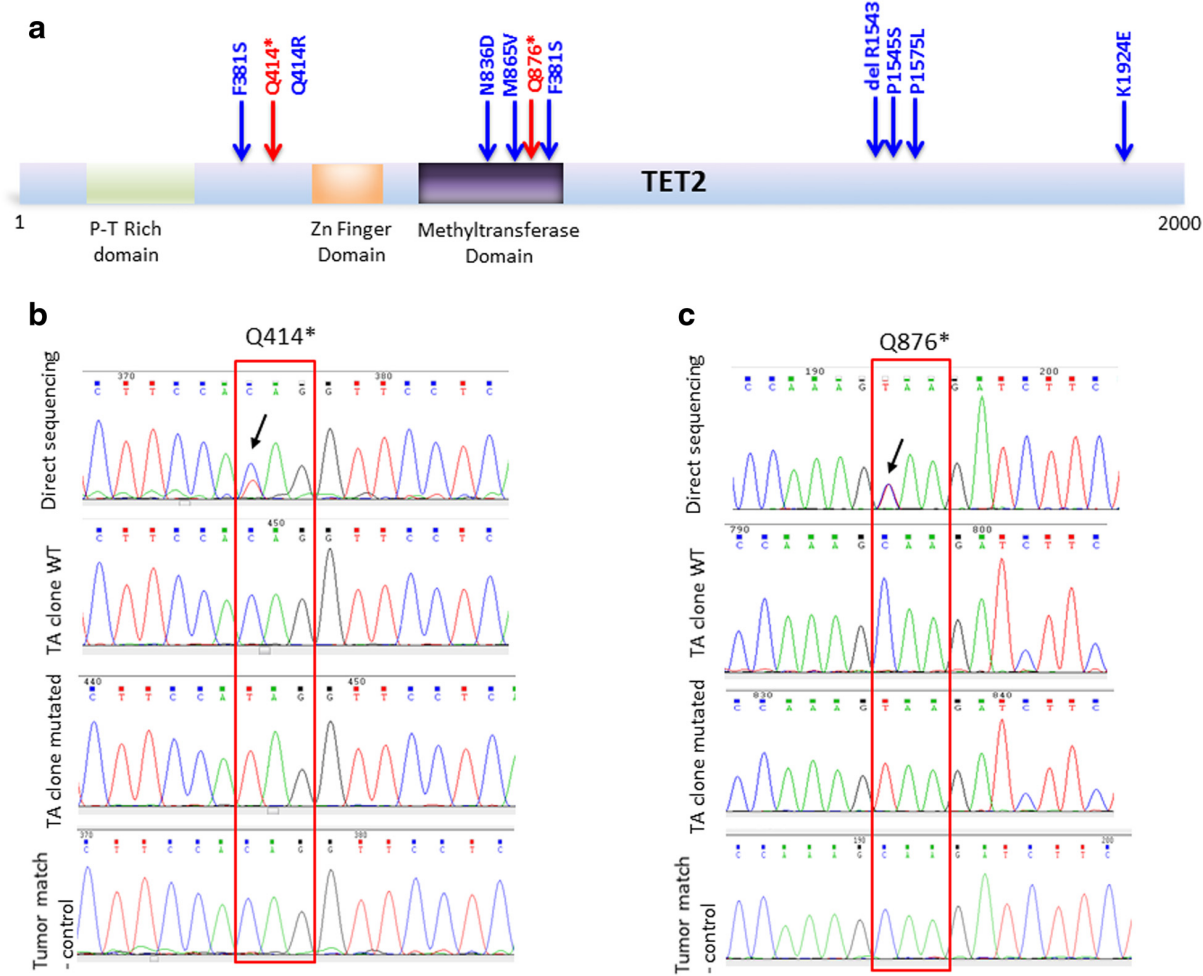
**a** Schematic representation of TET2 protein and distribution of mutations found in ATL patients. (**b** and **c**) Somatic LOH Q414* and LOH Q876* were confirmed by direct sequencing and analyses of TA clones from high grade tumor and matched non-tumor of two ATL samples

**Fig. 4:**
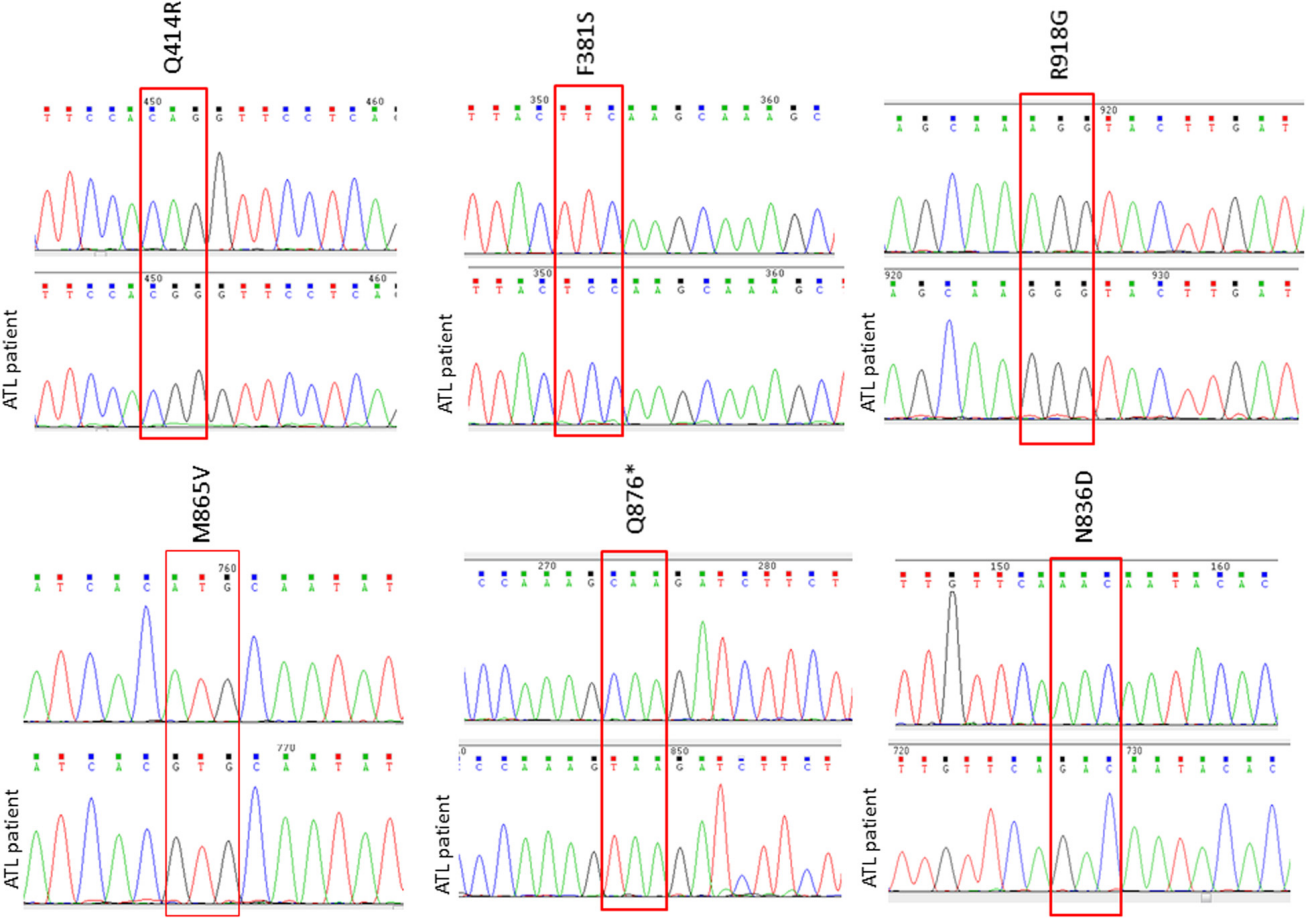
TET2 mutations were found in 6/18 ATL patients by TA-cloning and direct PCR sequencing

Ten-eleven translocation methylcytosine dioxygenase genes (TET1-3) are involved in DNA demethylation. Our investigations reveal mutation in the coding sequence of TET1 in 15 % (2/13). The mutation I1229M has been reported in the cosmic database. We also noticed the presence of TET1 single nucleotide polymorphism (snp rs3998860) I1123M. This snp has a global minor allele frequency (MAF) of 0.3067/1536 but was detected in all ATL samples tested (Additional file 1: Table S1). A larger cohort study is needed to confirm these data. No mutations were detected for TET3 (Additional file 1: Table S1). Interestingly, TET2 was mutated at a high frequency of 38 % (5/13) in ATL patients. These results are in line with the high rate of somatic TET2 inactivation observed in MDS, MPN, chronic myelomonocytic leukemia (CMML) and AML [28], and they suggest that TET2 may play an important role in ATL pathogenesis. TET2 LOH was found in two ATL patients with nonsense mutations at positions Q876* and Q414* (Fig.3a), two mutations previously reported in CML patients. For these two ATL patients, we PCR amplified the TET2 region overlapping these mutations and cloned and sequenced five clones for high tumor grade and matched samples. ATL12 DNA was extracted from a high grade lymph node biopsy from megakaryocytes as a tumor negative control. For ATL11 DNA was extracted from high proviral load (high grade tumor sample) samples before therapy and control sample DNA obtained after complete remission. Proviral loads were confirmed by quantitative real time RT-PCR for all samples (Fig. 1). Both mutations, Q876* and Q414*, were somatic mutations found in TET2 of ATL cells and not detected in control samples (Fig.3b and c). We then analyzed an additional 18 acute ATL patients by direct PCR, cloning and sequencing and found 6/18 (30 %) with the mutation (Fig. 4). Among missense ATL mutations only a mutation in position Q414R has previously been reported, although it was Q414L (COSM1618223). Of note, we found another unrelated ATL patient with a Q876* mutation suggesting this may represent a frequently mutated region for ATL (Fig. 4).

## Discussion

In this study we report a high frequency of TET2 missense mutations (8/31 (25 %)) and LOH of TET2 (3/31 (10 %)) in acute ATL patients. These data suggest that TET2 may be involved in HTLV-I pathogenesis and warrant additional studies. Studies have shown that the TET2 mutation results in global low levels of 5hmC compared with normal controls, supporting a functional relevance of TET2 mutations in leukemogenesis. Activating mutations of IDH1/2 have been shown to be mutually exclusive with mutations of TET2 [29]. Although mutations of IDH1/IDH2 have the same final epigenetic effect as TET2 inactivation, mainly a global promoter hypermethylation, mutation in IDH1/2 was not observed in any ATL samples tested here. Consistent with this notion, increased methylation of CDKN2A promoter has been associated with the progression of ATL disease [30]. Wilms tumor (WT1) mutant AML patients have reduced 5hmC levels similar to the TET2/IDH1/IDH2 mutant in AML, suggesting that WT1 may also play an important role in control of the epigenome [31]. WT1 and TET2 interact with one another. Although there are no reports regarding the genetic status of WT1 in ATL cells, we have previously shown PI3K-dependent cytoplasmic retention and inactivation of WT1 in HTLV-I transformed cells [32]. It will be interesting to evaluate the role of cytoplasmic WT1 in the regulation of TET2 functions in HTLV-I transformed T cells. Although a number of genes have been shown to be hypo- or hypermethylated in ATL cells, a direct implication of these genes in cellular transformation and/or ATL pathogenesis is lacking. In this study, we also found LOH in 1/13 ATL patient for MLL3. ASCOM-MLL3 has a redundant but crucial role in transactivation of p53 and participates in DNA damage-induced expression of p53-targeted genes [33]. Notably, p53 transcriptional functions are impaired in ATL patients in the absence of genetic mutations in p53 [34] and the possibility that loss of MLL3 participates in this process for some ATL patients warrants future studies.

## Conclusions

In summary, this report used both next generation sequencing and classic direct sequencing methods to identify mutations of epigenetic regulators in freshly isolated uncultured ATL samples. This study identifies for the first time mutations in multiple genes involved in the maintenance of the epigenome. Notably, a high frequency of mutation was detected in the TET2 gene with the presence of nonsense mutations leading to LOH in ATL patients. Our data suggest that TET2 and possibly MLL3 LOH may be involved in ATL pathogenesis and larger clinical correlative studies will now be needed to assess the effect on prognosis, diagnosis, and treatment of ATL.

### Key points

- Somatic mutations in TET2 and MLL3 in HTLV-I-associated human adult T cell leukemia, ATL.
- Inactivation of MLL3 and TET2 may play an important role in HTLV-I-induced ATL.

## Additional files

Additional file 1: Table S1. Novel mutations identified in epigenetic regulators EZH1, EZH2, EED, SUZ12, DNMT1, DNMT3A, DNMT3B, TET1, TET2, TET3, IDH1/2, MLL, MLL2, MLL3, MLL4 and ASXL1 in ATL patients. The mutations previously reported as SNPs are not included. Novel mutations are labeled in blue (missense) or in red (nonsense). Samples 1–7, 10 and 11 were isolated from patients diagnosed with acute ATL. Samples 8, 9, 12 and 13 were isolated from high proviral load tissues (Fig. 1) from patients diagnosed with lymphoma ATL. (PDF 231 kb) Additional file 2: Table S2. Sequence alignment of MLL3 gene and genome regions of very high homology found in chromosome 1, 2, 3, 13 and 21. Nucleotide differences specific to MLL3 are highlighted in red. TGA snp rs200662726 correspond to chromosome 13. (PDF 177 kb)
