# Erratum to: ‘Mutation of epigenetic regulators TET2 and MLL3 in patients with HTLV-I-induced acute adult T-cell leukemia’

**DOI:** 10.1186/s12943-016-0504-8

**Published:** 2016-03-02

**Authors:** Chien-Hung Yeh, Xue Tao Bai, Ramona Moles, Lee Ratner, Thomas A. Waldmann, Toshiki Watanabe, Christophe Nicot

**Affiliations:** 1 Department of Pathology, Center for Viral Oncology, University of Kansas Medical Center, 3901 Rainbow Boulevard, Kansas City, KS 66160 USA; 2 Department of Medicine, Division of Molecular Oncology, Washington University School of Medicine, Saint Louis, MO 63110 USA; 3 Lymphoid Malignancies Branch, Center for Cancer Research, National Institutes of Health, Building 10, Room 4 N/115, 10 Center Drive, Bethesda, MD 20892 USA; 4 Department of Medical Genome Sciences, University of Tokyo, Tokyo, Japan

Unfortunately, the original version of this article [1] contained an error. The author Toshiki Watanabe’s first name and surname were placed in the wrong order. Here we have included them in the correct order. There will be an update to go alongside this erratum in correcting this error.
