# Supplementary material for: Mutation of epigenetic regulators TET2 and MLL3 in patients with HTLV-I-induced acute adult T-cell leukemia
Source: Mol Cancer. 2016 Feb 16;15:15. doi: 10.1186/s12943-016-0500-z (PMC4754821; doi:10.1186/s12943-016-0500-z)
Supplement: Additional file 1: Table S1. — Novel mutations identified in epigenetic regulators EZH1, EZH2, EED, SUZ12, DNMT1, DNMT3A, DNMT3B, TET1, TET2, TET3, IDH1/2, MLL, MLL2, MLL3, MLL4 and ASXL1 in ATL patients. The mutations previously reported as SNPs are not included. Novel mutations are labeled in blue (missense) or in red (nonsense). Samples 1–7, 10 and 11 were isolated from patients diagnosed with acute ATL. Samples 8, 9, 12 and 13 were isolated from high proviral load tissues (Fig. 1) from patients diagnosed with lymphoma ATL. (PDF 231 kb) [file 12943_2016_500_MOESM1_ESM.pdf]

Table 1

	ATL 1	ATL 2	ATL 3	ATL 4	ATL 5	ATL 6	ATL 7	ATL 8	ATL 9	ATL10	ATL11	ATL12	ATL13
**EZH1/2**	wt	wt	wt	wt	wt	wt	wt	wt	wt	wt	wt	wt	wt
**EED**	wt	wt	wt	wt	wt	wt	wt	wt	wt	wt	wt	wt	wt
**SUZ12**	wt	wt	wt	wt	V68A<br>P74S	wt	wt	wt	wt	wt	wt	wt	wt
**DNMT1 Iso-a**	wt	wt	wt	wt	T161A<br>P153S<br>R151S<br>M122K	wt	wt	wt	wt	wt	wt	wt	wt
**DNMT3A iso-b**	wt	wt	wt	wt	wt	I103S<br>V107G	wt	wt	wt	wt	wt	wt	wt
**DNMT3B Iso-1**	wt	wt	N442K	wt	wt	wt	wt	wt	wt	wt	wt	wt	N442K
**TET1**	I1123M	I1123M	I1123M	I1123M<br>K931R	I1123M	I1123M	I1123M	I1123M<br>I1229T	I1123M	I1123M	I1123M	I1123M	I1123M
**TET2**	wt	wt	wt	wt	wt	del R1543<br>P1544S	wt	K1924E	del R1543<br>P1575L	wt	Q876*	Q414*	wt
**TET3**	wt	wt	wt	wt	wt	wt	wt	wt	wt	wt	wt	wt	wt
**IDH1/2**	wt	wt	wt	wt	wt	wt	wt	wt	wt	wt	wt	wt	wt
**MLL**	wt	wt	wt	wt	wt	S2511F		wt	wt	wt	wt		wt
**MLL2**	wt	wt	wt	wt	wt	T698I	wt	wt	wt	wt	wt	wt	wt
**MLL3**	wt	wt	R284Q	wt	R904*	I3590L	wt	V1089L	K433T	wt	wt	wt	wt
**MLL4**	wt	wt	wt	wt	P635R	G378E<br>E382K	wt	wt	G491S<br>P635R	wt	wt	wt	wt
**ASXL1**	wt	wt	wt	V1092M	S1166R	wt	wt	wt	wt	wt	wt	wt	wt
