# Supplementary material for: Mutation of epigenetic regulators TET2 and MLL3 in patients with HTLV-I-induced acute adult T-cell leukemia
Source: Mol Cancer. 2016 Feb 16;15:15. doi: 10.1186/s12943-016-0500-z (PMC4754821; doi:10.1186/s12943-016-0500-z)
Supplement: Additional file 2: Table S2. — Sequence alignment of MLL3 gene and genome regions of very high homology found in chromosome 1, 2, 3, 13 and 21. Nucleotide differences specific to MLL3 are highlighted in red. TGA snp rs200662726 correspond to chromosome 13. (PDF 177 kb) [file 12943_2016_500_MOESM2_ESM.pdf]

Table 2

```
MLL3      GCCAGGTCTCTTAATACTTATCTAAGGAAGTGTTTGTGTAACATTTAATAAAATGTTTTA
Chr21     GCCAGGTCTTTTAATACTTATCTAAAGAAGTGTTTGTATAACATTTAATAAAATGTTTTA
Chr1-1    GCCAGGTCTCTTAATACTTATCTAAAGAAGTGTTTGTATAACATTTAATAAAATGTTTTA
Chr1-2    GCCAGGTCTCTTAATACTTATCTAAAGAAGTGTTTGTATAACATTTAATAAAATGTTTTA
Chr1-3    GCCAGGTCTCTTAATACTTATCTAAAGAAGTGTTTGTATAACATTTAATAAAATGTTTTA
Chr2-1    GCCAGGTCTCTTAATACTTATCTAAAGAAGTGTTTGTATAACATTTAATAAAATGTTTTA
Chr2-2    -CCAGGTTTCTTAATACTTATCTAAAGAAGTGTTTGTATAACATTTAATAAAATGTTTTA
Chr13     GCCAGGTCTCTTAATACTTATCTAAAGAAGTGTTTGTATAACATTTAATAAAATGTTTTA
           ****** * *************** *********** **********************

MLL3      TCTCAGTGGCATTTGGATTTAAAAATTATTTTGGTCTGTCACAGAATGTTGACTTTTCCC
Chr21     TCTCAGTGGCATTTGGATTTAAAAATTATTTTGGGCTGTCACAGAATGTTGACTTTTCCT
Chr1-1    TCTCAGTGGCATTTGGATTTAAAAATTATTTTGGGCTGTCACAGAATGTTGACTTTTCCT
Chr1-2    TCTCAGTGGCATTTGGATTTAAAAATTATTTTGGGCTGTCACAGAATGTTGACTTTTCCT
Chr1-3    TCTCAGTGGCATTTGGATTTAAAAATTATTTTGGGCTGTCACAGAATGTTGACTTTTCCT
Chr2-1    TCTCAGTGGCATTTGGATTTAAAAATTATTTTGGGCTGTCACAGAATGTTGACTTTTCCT
Chr2-2    TCTCAGTGGCATTTGGATTTAAAAATTATTTTGGGCTGTCACAGAATGTTGACTTTTCCT
Chr13     TCTCAGTGGCATTTGGATTTAAAAATTATTTTGGGCTGTCACAGAATGTTGACTTTTCTT
          ********************************** ***********************

MLL3      AATCTGTTACATAGGGCCGTGGGTCTGGATTTCCAGGAAAGCGGAGACCTCGAGGTGCAG
Chr21     AATCTGTTACATAGGGCCATGGGTCTGGATTTCCAAGAAAGCGGAGACCTCGAGGTGCAG
Chr1-1    AATCTGTTACATAGGGCCATGGGTCTGGATTTCCAGGAAAGCGGAGACCTCGAGGTGCAG
Chr1-2    AATCTGTTACATAGGGCCATGGGTCTGGATTTCCAGGAAAGCGGAGACCTCGAGGTGCAG
Chr1-3    AATCTGTTACATAGGGCCATGGGTCTGGATTTCCAGGAAAGCGGAGACCTCGAGGTGCAG
Chr2-1    AATCTGTTACATAGGGCCATGGGTCTGGATTTCCAGGAAAGCGGAGACCTCGAGGTGCAG
Chr2-2    AATCTGTTACATAGGGCCATGGGTCTGGATTTCCAGGAAAGCGGAGACCTCGAGGTGCAG
Chr13     AATCTGTTACATAGGGCTGTGGGTCTGGATTTCCAGGAAAGCGGAGACCTCGAGGTGCAG
          *****************  **************** ************************

MLL3      GACTGTCGGGGCGAGGTGGCCGAGGCAGGTCAAAGCTGAAAAGTGGAATCGGAGCTGTTG
Chr21     GACTGTCGGGGCGAGGTGGCCGAGGCAGGTCAAAGCTGAAAAGTGGAATCGGAGCTGTTG
Chr1-1    GACTGTCGGGGCGAGGTGGCCGAGGCAGGTCAAAGCTGAAAAGTGGAATCGGAGCTGTTG
Chr1-2    GACTGTCGGGGCGAGGTGGCCGAGGCAGGTCAAAGCTGAAAAGTGGAATCGGAGCTGTTG
Chr1-3    GACTGTCGGGGCGAGGTGGCCGAGGCAGGTCAAAGCTGAAAAGTGGAATCGGAGCTGTTG
Chr2-1    GACTGTCGGGGCGAGGTGGCCGAGGCAGGTCAAAGCTGAAAAGTGGAATCGGAGCTGTTG
Chr2-2    GACTGTCGGGGCGAGGTGGCCGAGGCAGGTCAAAGCTGAAAAGTGGAATCGGAGCTGTTG
Chr13     GACTGTCGGGGTGAGGTGGCCGAGGCAGGTCAAAGCTGAAAAGTGGAATCGGAGCTGTTG
          *********** ************************************************

MLL3      TATTACCTGGGGTGAGGCTTGCTTCATGTATATTTTCTCTAATCTAAATGTCAGTTAATG
Chr21     TATTGCCTGGGGTGAGGCTTGCTTCATGTATATTTTCTCTAATCTAAATGTCAGTTAATG
Chr1-1    TATTGCCTGGGGTGAGGCTTGCTTCATGTATATTTTCTCTAATCTAAATGTCAGTTAATG
Chr1-2    TATTGCCTGGGGTGAGGCTTGCTTCATGTATATTTTCTCTAATCTAAATGTCAGTTAATG
Chr1-3    TATTGCCTGGGGTGAGGCTTGCTTCATGTATATTTTCTCTAATCTAAATGTCAGTTAATG
Chr2-1    TATTGCCTGGGGTGAGGCTTGCTTCATGTATATTTTCTCTAATCTAAATGTTAGTTAATG
Chr2-2    TATTGCCTGGGGTGAGGCTTGCTTCATGTATATTTTCTCTAATCTAAATGTCAGTTAATG
Chr13     TATTGCCTGGGGTGAGGCTTCCTTCATGTATATTTTCTCTAATCTAAATGTCAGTTAATG
          **** *************** ****************************** ********

MLL3      ATGAAAATCTCATAGCAAGTTATTTTGATCTTAAGAGTCATATAAATAGGTCAAAATGTT
Chr21     ATGAAAATCTCATAGCAAGTTATTTTGAT-----GAGTCATATAAATAGGTCAAAATGTT
Chr1-1    ATGAAAATCTCATAGCAAGTTATTTTGAACTTAAAAATCATATAAATAGGTCAAAATGTT
Chr1-2    ATGAAAATCTCATAGCAAGTTATTTTGAACTTAAAAATCATATAAATAGGTCAAAATGTT
Chr1-3    ATGAAAATCTCATAGCAAGTTATTTTGAACTTAAAAATCATATAAATAGGTCAAAATGTT
Chr2-1    ATGAAAATCTCATAGCAAGTTATTTTGAACTTAAAAGTCATATAAATAGGTCAAAATGTT
Chr2-2    ATGAAAATCTCATAGCAAGTTATTTTGAACTTAAAAGTTATATAAATAGGTCAAAATGTT
Chr13     ATGACAATCTCATAGCAAGTTATTTTGAACTTAAGAGTTATATAAATAGGTCAAAATGTT
          **** ***********************       * * *********************

MLL3      TATTTTACTGTCCTACTTTGCTTTTTTTTTT---------GAGCCTCTGGTTATGTTTTC
Chr21     TATTTTACTGTCCTACTTT-------TTTTT---------GAGCCTCTGGTTACATTTTC
Chr1-1    TATTTTACTGTCCTACTTTGCTTTTTTTTTTTTTTT----GAGCCTCTGGTTACGTTTTC
Chr1-2    TATTTTACTGTCCTACTTTGCTTTTTTTTTTTTTTT----GAGCCTCTGGTTACGTTTTC
Chr1-3    TATTTTACTGTCCTACTTTGCTTTTTTTTTTTTTTTT--TGAGCCTCTGGTTACGTTTTC
Chr2-1    TATTTTACTGTCCTACTTTGCTTTTTTTTTTTTTTTTTTTGAGCCTCTGGTTACGTTTTC
Chr2-2    TATTTTACTGTCCTACTTTGCTTTTTTTTTTTTTT---TTGAGCCTCTGGTTACGTTTTC
Chr13     TATTTTACTGTTCTACTTTGCTTTTTTTTT----------GAGCCTCTGGTTACGTTTTC
          *********** *******       ****          *************  *****
```
